# Supplementary figures and images for: Gender-based disparities and biases in science: An observational study of a virtual conference
Source: PLoS One. 2023 Jun 7;18(6):e0286811. doi: 10.1371/journal.pone.0286811 (PMC10246795; doi:10.1371/journal.pone.0286811)

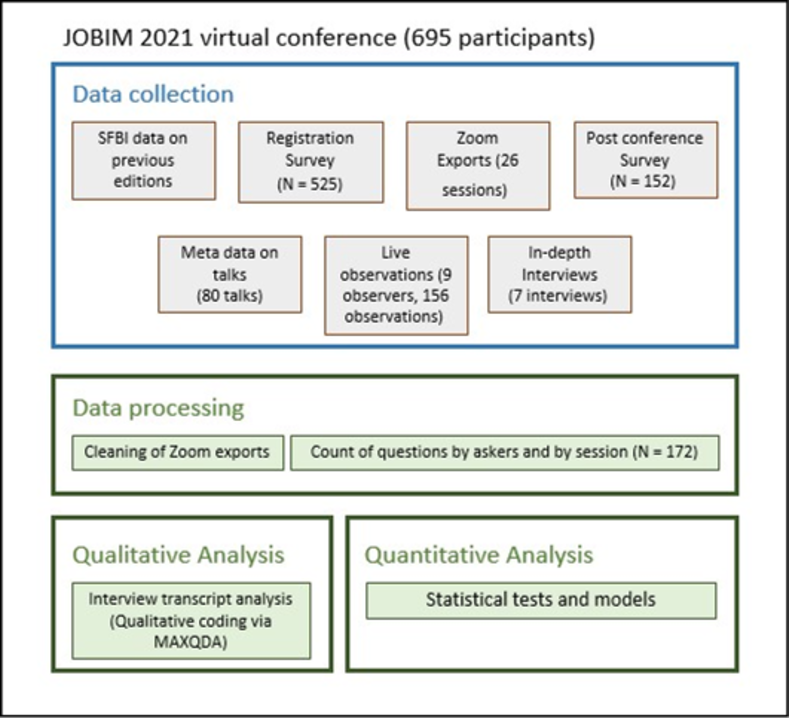

Supplement: S1 Fig — (TIF) [file pone.0286811.s001.tif]

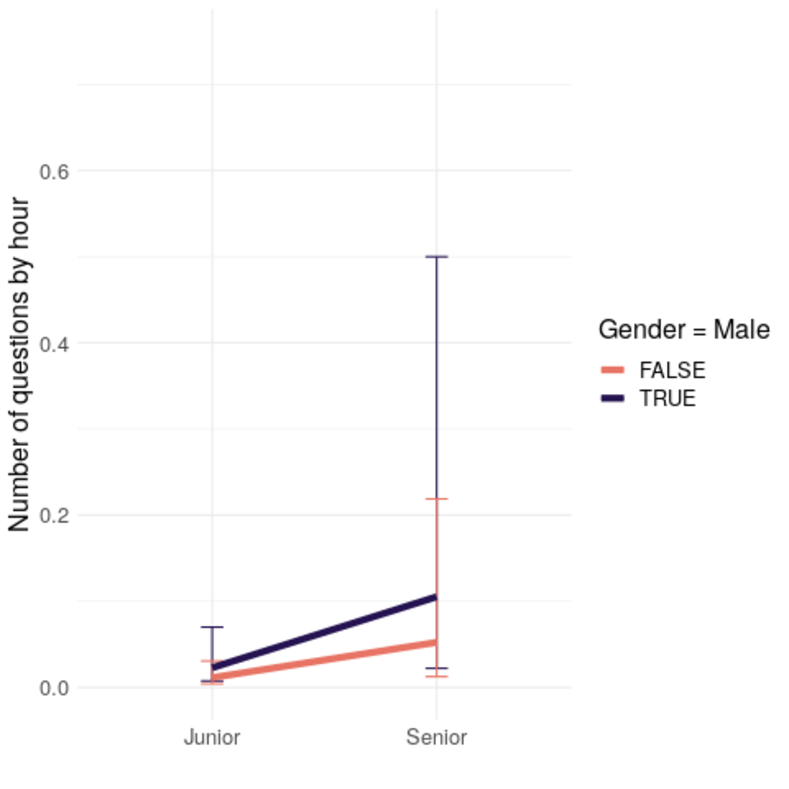

Supplement: S2 Fig — By senior, we refer to an attendee older than 35 and with a permanent position. By junior, we refer to an attendee younger than 35 and with a short term contract. (TIF) [file pone.0286811.s002.tif]

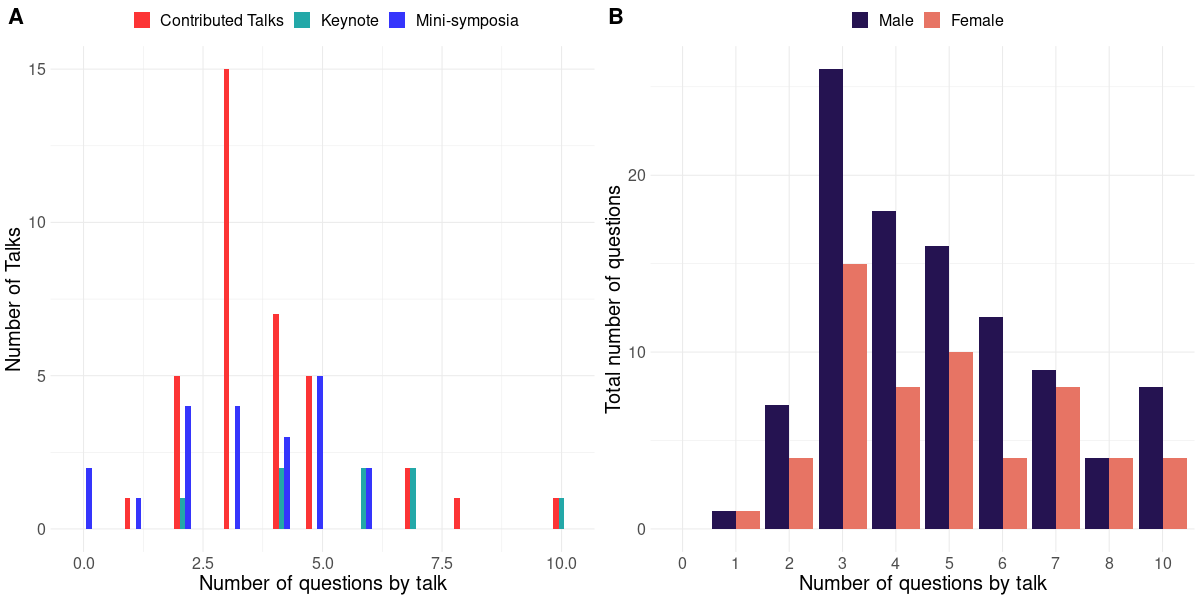

Supplement: S3 Fig — A) Histogram of the number of questions asked at the end of the talk by type of sessions (color coded), B) Total number of questions asked by gender (indicated by the color of the bars) with respect to the total number of questions asked at the end of the talk (used as a proxy for the duration of question session). (TIF) [file pone.0286811.s003.tif]

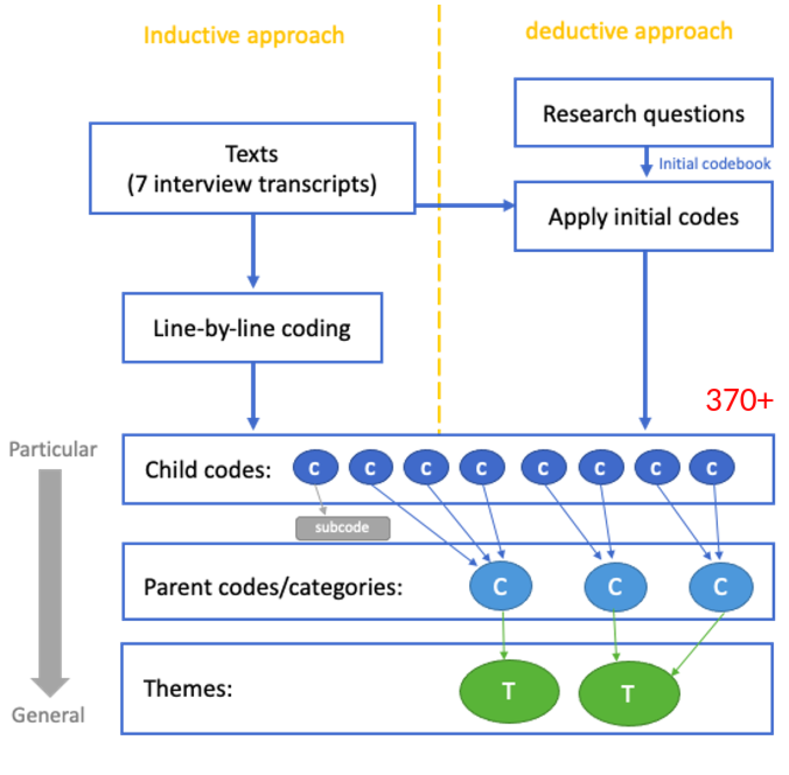

Supplement: S4 Fig — Each line of the transcript is assigned to a child code either derived from the research questions or created to represent the recurring topics. When all the transcripts have been processed, the resulting child codes are gathered into broader categories : parent codes. Finally parent codes are summarized in themes. (TIF) [file pone.0286811.s004.tif]

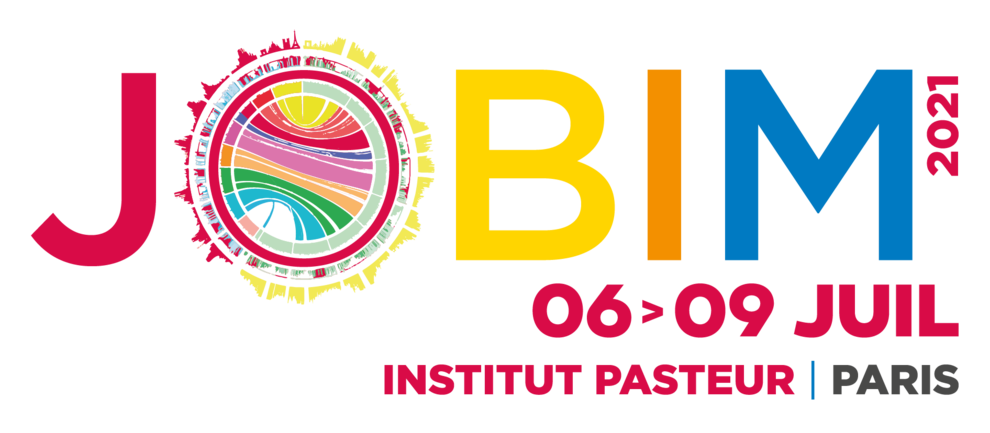

Supplement: S5 Fig — (PNG) [file pone.0286811.s005.png]
