# Supplementary material for: Gender-based disparities and biases in science: An observational study of a virtual conference
Source: PLoS One. 2023 Jun 7;18(6):e0286811. doi: 10.1371/journal.pone.0286811 (PMC10246795; doi:10.1371/journal.pone.0286811)
Supplement: S1 File — (PDF) [file pone.0286811.s006.pdf]

Planning for observations during the JOBIM 2021 Conference

JOBIM 2021 pilot project – "Gender Speaking Differences in Academia"

(for internal use only and used by the observers of this study and conference moderators)

I. Observational guidelines – instructions for collecting observational data

Background: This observational study is designed as part of the pilot project that focuses on gender speaking differences in academic. Since some studies have shown significant disparities between women and men in question-asking behaviours in academic seminar, we propose to perform the evaluation on gender speaking differences during the JOBIM 2021 Conference. Through this study, we hope to further discover the factors that contribute to gender-based biases in scientific conferences as well as the potential solutions for alleviating such inequality. Since the JOBIM 2021 Conference takes place virtually, the study will provide us with extra insights on disparities between men and women in question-asking behaviours in online settings. Our preliminary research on the existing literature has shown findings including female participants asking fewer questions than male participants in academic seminars. Through in-depth discussions with seminar participants and organisers at the Institut Pasteur, we acknowledge that such a phenomenon was commonly present in previous scientific seminars/conferences.
*The design of this planning is based on a set of hypotheses and research questions (attached in the Appendix[1])

Approach: In order to confirm the gender disparity in large scientific conferences and to delve deeper into the factors that possibly contribute to the unequal participation, we adopt an evident-based and ethnographic approach to this study. Participant observation is the key access to first-hand data. The following instructions for observations are designed to facilitate the observers' note-taking during each session and to record the data collection process.

Remark: The planning for observations is for internal use only and used by observers of this study and conference moderators. We recommended the viewers of this document to avoid sharing the detailed information with your colleagues or other conference attendees. This is to reduce the impact of this observation in the behaviours of the observees. We reply on your understanding and assistance to ensure the quality of our research.

II. Instructions for observations

The instructions are used by the observers of the study and conference moderators. The instructions for observing a session (keynote session/paradelle session) provide observational guideline for the following stages – 1) prior to the session, 2) during the session, and 3) after the session.

*Pilot testing of the observational sheet was performed. The testing confirmed that **at least 2 observers** are needed to observe one session simultaneously.

[1] Page 6

- Prior to the observation session:
  - Please make sure that you are familiar with the data collection procedure.
  - If possible, fill in some parts of the observational sheet (see the following section) based on information that you already have. For example: date, name of the observer, type of the session, name of the chairperson, name of the moderator(s), etc.

- During the session:
  - To observers: please fill in the following observational sheet basing on your observation. All fields are mandatory.
  - To moderators: please fill in the fields in blue in the following observational sheet, or provide the information in other ways (e.g., in a word document, or via emails).

We separate questions into two groups – interrupting questions (questions asked during the talk) and questions asked after the talk, although we acknowledge that for most of the sessions the attendees will not be able to interrupt the talks. Please be aware that a question can be a comment, a statement, or a statement that particularly solicits a response. Definitions of items in the observational sheet are listed as follows:

Date: the date of the session as DD/MM/YY, e.g., 09/07/21.
Time: the starting time of the talk as HHMM, e.g., 1430.
Observer: the name of the observer (the name will be anonymized).
Type of session: the typical types of sessions in the JOBOM Conference are keynote sessions and parallel sessions. If the observed session is neither of the two, please specify.
Title of session: the tile of the session (the title will be anonymized).
Speaker: the name of the speaker (the name will be anonymized).
Chairperson: the name of the chairperson (the title will be anonymized).
Moderator 1: the name of the first moderator (the name will be anonymized).
Moderator 2: the name of the second moderator (the name will be anonymized).
N_attendees: the number of attendees at the session.
N_male: the number of male attendees at the session.
N_female: the number of female attendees at the session.
Asker: the name of the attendee who asks the question (the name will be anonymized).
Gender: the observed gender of the person – male, female or N/A (if the information is not clear/available).
Question_time_start: the time at which the question starts as HHMM, e.g., 15:45.
Question_time_end: the time at which the question ends as HHMM, e.g., 15:46.
Question type: the question should be categorized as 1) Challenges, 2) Clarification, 3) Information-seeking, 4) Receptions, 5) Compliments / Containing compliments, or 6) Others______ / Not sure. Please use to the number to specify the type of the questions. One question can be classified into one or more of the above-mentioned categories.
Remarks: Please note down anything else that could be relevant to this study or provide complementary information.
N_questions: the total number of questions asked during the session.
N_male_asker: the total number of male askers.
N_female_asker: the total number of female askers.

1. Basic information of the session[2]

Date: ______________________ Time[3]: ____________________
Observer: ________________

Type of session: Keynote ☐ Parallel ☐ Other__________

Title of session: ____

Speaker: __________________ male ☐ / female ☐ / NA ☐

Chairperson: _________________ male ☐ / female ☐ / NA ☐

Moderator 1: _________________ male ☐ / female ☐ / NA ☐

Moderator 2: _________________ male ☐ / female ☐ / NA ☐

2. Attendees of the session

N_attendees: ☐ N_male: ☐ N_female: ☐

* Please calculate the number of attendees 15 minutes after the session has started

Name list of the attendees:

* Please extract the name list 15 minutes after the session has started

3. Question-answer behaviours

- Interrupting questions (during the talk)

Number	Asker	Gender	Question_ time_start	Question_ time_end	Question_ type[4]	Remarks
Question_1		male☐ / female☐ / NA☐				
Question_2		male☐ / female☐ / NA☐				

[2] All identifications of gender in this observational sheet refer to "observed gender".
[3] Starting time of the observation.
[4] Categorizations: 1) Challenges; 2) Clarification; 3) Information-seeking; 4) Receptions; 5) Compliments / Containing compliments; 6) Others______ / Not sure

- Questions asked after the talk

Number	Asker	Gender	Question_ time_start	Question_ time_end	Question_ type	Remarks
Question_1		male☐ / female☐ / NA☐				
Question_2		male☐ / female☐ / NA☐				
Question_3		male☐ / female☐ / NA☐				
Question_4		male☐ / female☐ / NA☐				

- Total questions asked

N_questions: [ ] N_male_asker: [ ] N_female_asker: [ ]

4. Questions in the chat

- Messages in the public chat box with details, including times and names (copy all messages from the chat box in the end of the session):

- Messages sent privately to the moderators with details, including times and names:

- After the session:
  - Please make sure the competed observational sheets and collected data are stored in a safe place.
  - Send all the information to the JOBIM pilot study team (jobim-project@pasteur.fr) once you finish your tasks as an observer/moderator.

III. Contact information

For questions and more information, please contact contact us at jobim-project@pasteur.fr. To get in touch with individual researchers of the project, please refer to the following contact list:

Junhanlu Zhang (junhanlu.zhang@pasteur.fr)
Eng. Rachel Torchet (rachel.torchet@pasteur.fr)
Dr. Hanna Julienne (hanna.julienne@pasteur.fr)

An observational training session will soon be organized for observers.
A Q&A session may be organized for moderators too, depending on the requests we receive. We will keep you updated once such sessions are planned.

Appendix

**Hypotheses/research questions**	**Observational data**	**Remarks**
1. Do men ask more questions than other genders during keynote and poster sessions?	• Numbers of question asked	
	• Observed gender of the asker (male/female/NA)	
	• Name of the asker (if not anonymous)	For later use to compare the observed gender with the self-identified gender.
2. What is the gender ratio of the session and is it a factor that influence participants' question-asking behaviours?	• Total number of participants	
	• Number of participants by their observed genders (male/female/NA)	
	• Name list of the participants	For later use to compare the observed gender with the self-identified gender.
3. Are interrupting questions[5] more likely to be asked by men?	• Observed gender of the person who asks interrupting questions	
	• Name of the person who asks interrupting questions	For later use to compare the observed gender with the self-identified gender.
4. Are the first questions[6] more likely to be asked by men?	• Observed gender of the person who asks the first question	
	• Name of the person who asks the first question	For later use to compare the observed gender with the self-identified gender.
5. Do men take more time to ask questions than women and other genders do?	• Starting time of the question asked	
	• Ending time of the question asked	
	• Observed gender of the asker	
	• Name of the asker	For later use to compare the observed gender with the self-identified gender.
6. Do the genders of the speaker and moderator impact question-asking behaviours of the participants?	• Observed gender of the speaker	
	• Name of the speaker	For later use to compare the observed gender with the self-identified gender.
	• Observed gender of the moderator	
	• Name of the moderator	For later use to compare the observed gender with the self-identified gender.
7. Are there emotional differences in question-asking behaviours based on the gender of the asker?	• Categorization of each question asked	- Challenges<br>- Clarification<br>- Information-seeking<br>- Receptions<br>- Compliments / Containing compliments<br>- Others / Not sure

[5] Questions that interrupt the speaker during his/her talk.
[6] First questions asked after a talk or presentation.
