# Supplementary material for: Gender-based disparities and biases in science: An observational study of a virtual conference
Source: PLoS One. 2023 Jun 7;18(6):e0286811. doi: 10.1371/journal.pone.0286811 (PMC10246795; doi:10.1371/journal.pone.0286811)
Supplement: S2 File — (PDF) [file pone.0286811.s007.pdf]

Interview Consent Form

Research project title
JOBIM 2021 Pilot Project - Gender Speaking Differences in Academia

Research investigators
Junhanlu Zhang, Rachel Torchet & Hanna Julienne

Participant's name: ______________________   Interview Date: _____ /_____ /________

Description of the project

In the JOBIM 2021 conference, we are launching a pilot project on gender speaking differences in academia (https://research.pasteur.fr/en/project/jobim-2021-pilot-project-gender-speaking-differences-in-academia/). Through this evidence-based and mixed-method study, we intend to answer the following question: how to create conditions for gender-equal expression in scientific conferences?

Thank you for agreeing to be interviewed as part of the above research project. This interview will take approx. 30-45 minutes. We do not anticipate that there are any risks associated with your participation. However, sensitive personal information may be involved during the interview, and you have the rights to stop the interview or withdraw from the research at any time.

Based on the GDPR regulations, this consent form is necessary for us to ensure that you understand the purpose of your involvement and that you agree the conditions of your participation. Would you therefore read the accompanying **information sheet** and sign this form to certify that you approve the following:

- The interview will be recorded, and a transcript will be produced.
- You will have the access to the transcript as well as the opportunity to correct errors.
- The transcript of the interview will be analysed by Junhanlu Zhang as research investigator.
- Access to the interview recording and transcript will be limited to research investigators of this project – Junhanlu Zhang, Rachel Torchet and Hanna Julienne.
- The information from your interview will be processed and published anonymously. Care will be taken to ensure that other information in the interview that could identify yourself is not revealed.
- The actual recording will be deleted once the project is completed (by the end of the year 2021).
- Any variation of the conditions above will only occur with your further explicit approval.

By signing this consent form, I agree that:

- I am participating in this research project voluntarily, and I understand that I can stop the interview or withdraw from the project at any time.

- I understand that I can exercise other individual rights in compliance with the GDPR regulations – I can access the transcript, object to the processing of the interview data, and I also have the right to the portability of the interview data.

  To exercise any of these rights, I can simply contact the research team via email (jobim-project@pasteur.fr).

- I have carefully read the information sheet.

- I do not expect any benefit or payment for my participation.

- I understand that I can express myself freely and ask questions whenever I need to during the interview.

- I understand that I am free to contact research team (jobim-project@pasteur.fr) with any question I may have regarding this project in the future.

Participant's signature	Researcher's signature
Date signed	Date signed
_____/_____/________	_____/_____/________

Institut Pasteur

JOBIM 2021 06>09 JUIL INSTITUT PASTEUR | PARIS
