# Supplementary material for: Gender-based disparities and biases in science: An observational study of a virtual conference
Source: PLoS One. 2023 Jun 7;18(6):e0286811. doi: 10.1371/journal.pone.0286811 (PMC10246795; doi:10.1371/journal.pone.0286811)
Supplement: S3 File — (PDF) [file pone.0286811.s008.pdf]

Interview guide (with interview questions)

Research project title
JOBIM 2021 Pilot Project - Gender Speaking Differences in Academia

Research investigators
Junhanlu Zhang, Rachel Torchet & Hanna Julienne

This interview guide is developed to assist the interviews as part of the JOBIM 2021 Pilot Project. The guide consists of guidelines throughout three stages of the interview procedure – preparation, interview, and post-interview, as well as interview questions covering three major areas – demographic information, experience with academic conferences, and gender-related questions in STEM. Basic information of the interviews is as following,

Type of interviews: semi-structured in-depth interviews
Interviewees: attendees of the JOBIM 2021 conference who agreed at the conference registration stage to contribute to this study
Interviewee sample size: 5-7 interviewees
Time of interviews: 30-45 minutes
Interview channel: videoconferencing platforms (e.g., Teams and Zoom)

Stage 1: Preparation

- Invite potential interviewees to join the interviews and schedule the time/date once the interview is confirmed.
- Send an interview reminder to the interviewee with the consent form for both parties to sign 1-3 days prior to the agreed interview time.
- If applicable, pay attention to the interviewee's registration and post-survey data to adapt the interview accordingly.
- Prepare the equipment (e.g., notebook and pen) to take complimentary notes and to make backup recording (e.g., recording pen or phone) during the interview.

Stage 2: Interview

<table>
<tr>
<td>Introduction</td>
<td colspan="2">- Introduce the project and the purpose of the interview.

- Emphasize on the confidentiality of the interview, and double check with the interview if they understand the information provided on the consent form.

- Let the interviewee know that they can interrupt to ask questions anytime during the interview. If there is any question that makes the interviewee uncomfortable, they can refuse to answer the question, or even stop the interview if necessary.

- Asked for permission to record and start recording.</td>
</tr>
<tr>
<td rowspan="2">Research questions</td>
<td>Demographic information</td>
<td>- Hong long have you been studying and/or working in the STEM fields?

- Which is your specialised area of work?

- What is your current institution?

- How settled are you in your current position?

- How is it like working in STEM in general?</td>
</tr>
<tr>
<td>Experience with academic conferences</td>
<td>- How often do you participate in academic conferences either in your professional field or in other fields?

- Is attending conferences important to you? Why?

- How do you like such an event based on your past experiences?

- What usually motivate you to attend a conference?

- How active are you in academic conferences? For example, do you ask questions?

- Is it usually easy for you to ask questions during the conference? Why?</td>
</tr>
</table>

		- Have you participated in online conferences since the pandemic started and how did you like them?<br><br>- Are online and offline conferences different in your opinion? What is your preference?<br><br>- What are your experiences when it comes to meetings and academic seminars? Is it easy to express yourself and how are your ideas received usually?
	Gender-related questions in STEM	- What are/were the biggest obstacles that you recognise throughout your career development?<br><br>- Based on your observation, do you see an inequality or imparity between men and women in the STEM fields?<br><br>- The existing literature often considers women as the minority and underprivileged population in STEM, do you agree? Why?<br><br>- What is your general impression on gender diversity and inclusion in STEM?<br><br>- Do you think your gender identity or sexual orientation matters in your work and/or professional life? If you do, in what way does it intervene your work and/or professional life?<br><br>- Have you experienced or observed any type of gender-based discrimination and harassment in conferences or in the workplace?

		- In your opinion, should we do more to reach gender diversity, equity, and inclusion in STEM? Any ideas or advice?
Conclusion	- Ask the interviewee if there is any question or anything to add.<br><br>- Stop the recording.<br><br>- Express appreciation, and let the interviewee know that they can get in touch in the future if there is anything that they would like to address after the interview. Provide contact information if needed.	

Stage 3: Post-interview

- Check notes and add necessary information (e.g., observations during the interview, clarification on notes, or reflections as the interviewer) as soon as possible after the interview is over.
- Check the quality of recording and make sure that the recording is kept in a secure place.
